# Age Differences in Speech Perception in Noise and Sound Localization in Individuals With Subjective Normal Hearing

**DOI:** 10.3389/fpsyg.2022.845285

**Published:** 2022-04-15

**Authors:** Tobias Weissgerber, Carmen Müller, Timo Stöver, Uwe Baumann

**Affiliations:** ^1^Audiological Acoustics, Department of Oto-Rhino-Laryngology, University Hospital Frankfurt, Goethe University Frankfurt, Frankfurt am Main, Germany; ^2^Department of Oto-Rhino-Laryngology, University Hospital Frankfurt, Goethe University Frankfurt, Frankfurt am Main, Germany

**Keywords:** speech perception, noise, sound localization, age, cognition

## Abstract

Hearing loss in old age, which often goes untreated, has far-reaching consequences. Furthermore, reduction of cognitive abilities and dementia can also occur, which also affects quality of life. The aim of this study was to investigate the hearing performance of seniors without hearing complaints with respect to speech perception in noise and the ability to localize sounds. Results were tested for correlations with age and cognitive performance. The study included 40 subjects aged between 60 and 90 years (mean age: 69.3 years) with not self-reported hearing problems. The subjects were screened for dementia. Audiological tests included pure-tone audiometry and speech perception in two types of background noise (continuous and amplitude-modulated noise) which was either co-located or spatially separated (multi-source noise field, MSNF) from the target speech. Sound localization ability was assessed and hearing performance was self-evaluated by a questionnaire. Speech in noise and sound localization was compared with young normal hearing adults. Although considering themselves as hearing normal, 17 subjects had at least a mild hearing loss. There was a significant negative correlation between hearing loss and dementia screening (DemTect) score. Speech perception in noise decreased significantly with age. There were significant negative correlations between speech perception in noise and DemTect score for both spatial configurations. Mean SRTs obtained in the co-located noise condition with amplitude-modulated noise were on average 3.1 dB better than with continuous noise. This gap-listening effect was severely diminished compared to a younger normal hearing subject group. In continuous noise, spatial separation of speech and noise led to better SRTs compared to the co-located masker condition. SRTs in MSNF deteriorated in modulated noise compared to continuous noise by 2.6 dB. Highest impact of age was found for speech perception scores using noise stimuli with temporal modulation in binaural test conditions. Mean localization error was in the range of young adults. Mean amount of front/back confusions was 11.5% higher than for young adults. Speech perception tests in the presence of temporally modulated noise can serve as a screening method for early detection of hearing disorders in older adults. This allows for early prescription of hearing aids.

## Introduction

The World Health Organization (WHO) estimates that approximately one third of all people over the age of 65 suffer from a hearing loss, although the number of unreported cases is likely to be even higher, as it usually takes time before a progressive hearing loss is diagnosed (World Health Organization, 2017). From 2008 to 2030 the age group of people aged 65 and older will increase in Germany by around one third due to demographic change (Statistische Ämter des Bundes und der Länder, 2011). For the whole European Union, the estimated increase in that age group for the same time span is about 45% (European Commission, 2020). This means that hearing loss in particular will become more important in the elderly. In addition, lifestyles in later life have changed considerably. Today, there are numerous options for living arrangements such as nursing homes or assisted living. In order to continue participating in social life, more and more elderly rely on telecommunication or events such as senior citizens’ meetings. The range of possible consequences of hearing impairment is wide, including social isolation and inability to work or psychosomatic disorders such as anxiety and depression (Garnefski and Kraaij, 2012).

Although hearing impairment has a major impact on the quality of life of the elderly. hearing loss in elderly subjects is frequently undetected and untreated (Völter et al., 2020). In a study population aged between 40 and 79 years with at least a mild hearing loss, only 6% were aware of any symptoms (Ramage-Morin et al., 2019).

It is known that speech perception in noise is impaired even in the presence of a mild hearing loss compared to age-matched normal-hearing individuals (Dubno et al., 1984). In the study of Dubno et al. (1984) it was also shown that older subjects with normal hearing (which was comparable to a younger subject group) showed decreased speech perception in noise for suprathreshold signal presentation.

Likewise, Meister et al. (2011) reported slightly degraded speech reception thresholds in quiet and continuous noise but substantial differences were found for modulated noise. It was reported by Füllgrabe (2013) that suprathreshold processing of temporal fine structure (TFS) declines with increasing age even when hearing sensitivity is (nearly) normal. In a subsequent study, Füllgrabe et al. (2015) showed decreased speech perception scores for consonants and sentences in noise in a study population aged older than 60 years with pure-tone thresholds matched to audiometrically normal-hearing younger (<30 years) adults. Furthermore, a correlation between consonant perception as well as speech perception in noise and sensitivity to TFS was shown. In concordance with Meister et al. (2011), a correlation between speech perception scores (consonants and sentences) and several cognitive measures was also reported by Füllgrabe et al. (2015).

In Summary, increasing age adversely affects the processing of both TFS and slowly varying envelope (ENV), whereby a stronger correlation with speech perception in noise was found for TFS. Therefore, spatial release from masking (SRM) is reduced for speech stimuli in older subjects with or without hearing loss. This implies that aged subjects will have difficulties relative to young normal-hearing subjects when trying to understand speech in the presence of interfering sounds coming from different directions in space, as is common in everyday life (Moore, 2021).

Therefore, the aim of the present study was to investigate speech perception under complex noise conditions with multiple noise sources in a cohort of older persons. Speech reception thresholds (SRTs) were assessed in two types of background noise (continuous and amplitude-modulated noise) which was either co-located or spatially separated (four sound sources, multi-source noise field, MSNF) from the target speech (frontal presentation).

It was expected that impaired TFS and ENV processing as well as a potentially reduced SRM effect would show an impact on SRTs as a function of age. In addition, spatial hearing ability was evaluated based on the accuracy of sound localization obtained for broadband noise stimuli. Screening of cognitive performance was assessed to identify potentially deteriorated results that might correlate with the hearing test battery outcomes. Finally, results from a cohort of seniors with no self-reported hearing loss (i.e., subjectively no known symptoms of hearing loss, normal to mild hearing loss) were compared with data obtained in previous studies using the same test setup in young adults with normal hearing (Weissgerber et al., 2017).

## Materials and Methods

### Subjects

The study comprised a total of 40 subjects (28 female, 12 male). The subjects were aged between 60.1 and 89.7 years (mean age: 69.3 ± 7.1 years, mean age of the female subjects: 69.6 ± 7.1 years, mean age of the male subjects: 68.6 ± 7.5 years).

Subjects were recruited for participation via flyers and an advertisement at the grounds of the University Hospital Frankfurt. The three inclusion criteria mentioned in that advertisement were (1) aged 60 or higher, (2) no subjective awareness of any hearing problems, and (3) no use of hearing aids. Each of the subjects was a native German speaker, as the speech perception tests were conducted in German. The study was approved by the Ethics Committee of the Department of Medicine of the Goethe University in Frankfurt am Main, Germany (No. 164/13).

Before performing the tests, an ear inspection was performed and a tympanogram was obtained for each ear to exclude study candidates with eventual conductive hearing loss. The study tests required ~3 h per subject and were each conducted on 1 day. The order of study tests was randomized.

### Screening for Dementia

The DemTect (Kessler et al., 2000) was used to check for a potential onset of dementia. The DemTect consists of five subtests, which are carried out in the form of a survey. A list of ten words is read out to the subject and then immediately queried. Afterward, the same list is again read out and queried again. At the end of the test the word list has to be repeated by the subject without being read out again in order to test verbal memory. Furthermore, there is a subtest on intellectual flexibility in which numbers have to be converted into text and vice versa. Finally, there is a subtest for word fluency, in which the subject has to list things that can be bought in the supermarket within 1 min. A further subtest on verbal memory and attention follows with the reproduction of a sequence of numbers read out in reverse order.

The results of the individual subtests are converted into age-corrected test scores, then summed up and expressed as a DemTect score (max. 18 points). The resulting test scores are independent of age and educational level and thus provide information on whether cognitive performance is age-appropriate (DemTect score: 13–18 points), slightly impaired (DemTect score: 9–12 points) or whether dementia is suspected (DemTect score: ≤8 points) (Kalbe et al., 2004). The duration of the DemTect is ~8–10 min.

### Pure-Tone Audiometry

Pure-tone audiometry was performed in a sound-attenuated room to determine the subjects’ individual hearing thresholds. Air conduction hearing thresholds were determined for pure-tones from 125 to 8,000 Hz for each ear of each subject using calibrated headphones. Bone conduction hearing thresholds were not determined because middle ear pathologies were excluded in advance both by patient history and by tympanometry. The pure-tone average (PTA) hearing loss was determined from the frequencies 500 Hz, 1, 2, and 4 kHz (PTA_4). Furthermore, a pure-tone average hearing loss for high frequencies was calculated as mean hearing loss of the frequencies 4, 6, and 8 kHz (PTA_high).

### Speech Perception in Noise

Speech tests were conducted for different types of background noise in two spatial loudspeaker configurations to simulate everyday listening situations. The measurements were conducted in an anechoic chamber with dimensions 4.1 m × 2.6 m × 2.1 m (length × width × height). The system for sound playback consisted of 128 loudspeakers arranged in a rectangular array in the horizontal plane at a height of 1.20 m, which corresponded approximately to the ear height of the seated subjects. Further detailed information on the playback system is given in Weissgerber et al. (2015).

Speech reception thresholds (SRTs) in noise were determined with the German matrix test [Oldenburg Sentence Test, OLSA, Wagener et al. (1999)]. Each test list consisted of 20 sentences, which contained a noun, verb, numeral, adjective, and object. Noise level was kept constant at 65 dB SPL and speech level was set adaptively according to the number of words perceived correctly. Speech levels automatically increased when two or fewer words were perceived correctly and decreased when more than two words were correct. The step sizes for this adaptive procedure decreased with the number of inflection points as suggested by Brand and Kollmeier (2002). The result of the OLSA test is the SRT for 50% correct word understanding. Speech signal was always presented from the same direction of 0° frontally at a distance of 1.75 m from the subject. Four adjacent speakers of the playback system were used to obtain a sound pressure level with negligible distortion at the subject’s position (Weissgerber et al., 2017).

The test was conducted in a closed set mode, i.e., the subject had to select the perceived words of the sentence on a matrix presented on a touchscreen. In order to become familiar with the task, a training trial with 30 test sentences was performed with each subject before the study test began. Subsequently, four test runs of the OLSA were performed with each subject in random order, differing in noise type and spatial noise configuration.

Two types of noise were used. The speech-shaped “OlNoise” is a temporally continuous noise whose long-term spectrum matches that of the word material of the matrix test (Wagener et al., 1999). This continuous noise is used to simulate background noise with low temporal modulation, such as the noise of a vacuum cleaner or a fan. The other test stimulus was amplitude-modulated, speech-shaped, fluctuating noise according to Fastl (1987) and Fastl and Zwicker (2007). The spectral distribution of the amplitude-modulation reaches maximum values at 4 Hz, which is consistent with many spoken syllables of Western speech.

Two spatial noise configurations were tested. In the first condition, noise was presented from the same direction as speech signal (0°, condition S0N0).

**FIGURE 1:**
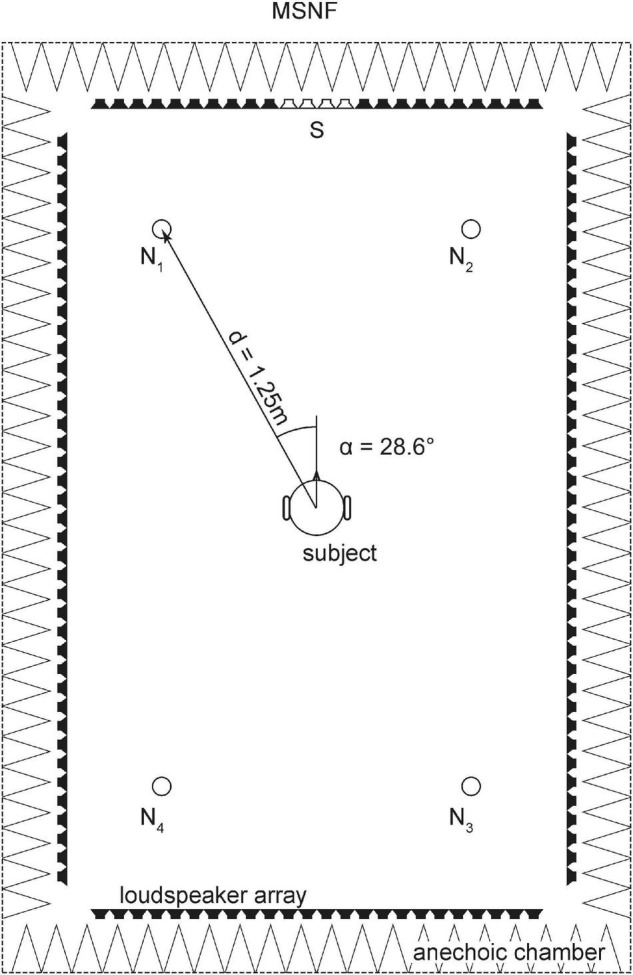
Loudspeaker array with 128 independent loudspeaker channels in rectangular shape mounted in the horizontal plane at 1.20 m for measurement of speech perception in noise. The speech signal (S) was generated from front loudspeakers in the 0°-position. Four virtual noise sources (N1-4) for multi-source noise field (MSNF) were created by wave field synthesis.

In the second condition, a diffuse noise (multi-source noise field, MSNF) was created by means of wave field synthesis (Berkhout, 1988). Four virtual noise sources were placed at the positions ±28.6° and ±151.4° with a distance of 1.25 m from the center of the subject’s head (see Figure 1). The four virtual noise sources were temporally uncorrelated. The MSNF speaker configuration was proposed by Rader et al. (2013) to simulate everyday conversational situations in noisy environments, such as conversations in a restaurant, etc.

In the study of Weissgerber et al. (2017) data obtained in young adults with normal hearing (*n* = 14, mean age: 26.4 ± 5.4 years, range: 22–37.3 years) using the identical test setup was shown. The criterion for normal hearing was a pure-tone hearing loss lower than 25 dB HL between 0.25 and 8 kHz. The results obtained in the cohort of older subjects in the present work were compared with the data from Weissgerber et al. (2017).

### Sound Localization

The test for sound localization took place in the same anechoic room with the same loudspeaker arrangement. LED chains with a total of 704 individual LEDs were mounted above the loudspeakers to indicate the direction of sound incidence.

The test stimulus was a white noise (high-pass filtered at 150 Hz) consisting of five pulses. Each pulse had a duration of 30 ms with a rise time of 3 ms followed by a pause of 70 ms [according to Seeber (2002)]. Before the test began, a blue LED lit up in front of the subject at the 0° position, at which the subject had to focus on. After hearing the test stimulus, the subject was first asked to indicate by means of a toggle switch that changed the LED color whether the sound was perceived from the front (red LED) or from behind (green LED). Subsequently, the subject should select the LED that corresponded to the perceived horizontal angle of incidence of the auditory event using a rotary encoder. The indication of a sound from behind was marked green *via* the toggle switch and mirrored to the front. Prior to the start of the test, a detailed introduction to the LED display system took place, as well as a training run in which each test loudspeaker was tested once. A total of seven test loudspeakers were tested between −60 and +60° (−59.2°, −42.1°, −21.2°, −2.5°, 16.8°, 42.1°, 59.2°) in front and back. Each of the 14 test loudspeakers was randomly selected five times (i.e., 70 trials) in order to measure the localization accuracy (mean error, i.e., deviation of presented angle and perceived angle) and uncertainty (dispersion of mean error) of localization. The test was performed in complete darkness. The duration of a whole test run was about 15 min.

The relative localization error was calculated for each subject by averaging the relative localization errors of each angle (both front and back). Furthermore, the percentage front-back confusions were calculated. Results were compared with data obtained in young adults with normal hearing (*n* = 9, mean age: 30.3 ± 6.1 years, pure-tone hearing loss lower than 25 dB HL for all test frequencies between 0.25 and 8 kHz) using the identical test setup (data unpublished so far).

### Speech, Spatial, and Qualities of Hearing Scale Questionnaire

All 40 senior subjects completed the validated German-language version of the “Speech, Spatial, and Qualities of Hearing Scale” (SSQ) questionnaire (Kießling et al., 2011) to assess their subjective hearing performance. The SSQ questionnaire is a standardized and validated questionnaire consisting of 49 questions on various listening situations, which the subject answers by marking his subjectively assessed listening ability on a Likert scale of 0–10 points (Gatehouse and Noble, 2004). The questions are divided into three sections. The first section contains questions on speech perception in a wide variety of everyday listening situations. The second section includes questions on spatial hearing. The third section focuses on listening quality, e.g., naturalness, clarity and identifiability of a speaker.

### Statistics

The collected data was processed and analyzed using the statistical program SPSS Statistics 27 (IBM, Armonk, NY, United States). The target variables of the various tests were checked for normal distribution. Since none of the target variables showed a normal distribution, further evaluation was carried out with non-parametric methods. The Spearman Rho test was used to examine the correlation between the test variables. For multiple comparisons *p*-values were adjusted using the Bonferroni-Holm method. Adjusted *p*-values < 0.05 were considered as statistically significant. Only adjusted *p*-values were given in the manuscript if not stated otherwise.

## Results

### Dementia Screening

**FIGURE 2:**
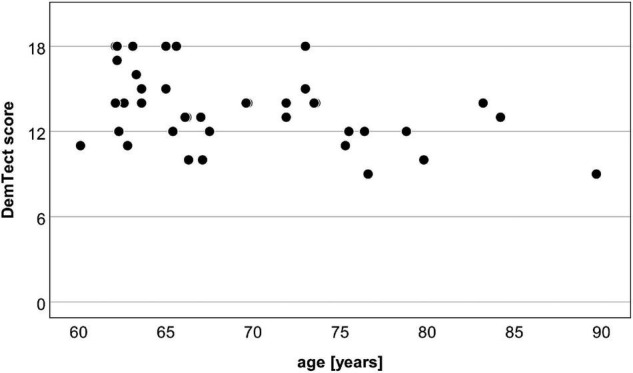
Scatter plot of age and DemTect scores of the 40 test subjects. Thirteen–eighteen points: age-appropriate cognitive performance; 9–12 points: slight impairment; ≤8 points: suspicion for dementia.

Individual results of the DemTect are shown in Figure 2. The median DemTect score of all subjects was 14.0 points (interquartile range IQR: 12.0 to 15.0 points). Sixty five percentage of the subjects had DemTect scores between 13 and 18 points, so that their cognitive performance can be classified as age-appropriate. The remaining 35% of the subjects had DemTect scores between 9 and 12 points and thus a mild cognitive impairment. None of the subjects had a DemTect score that indicated a suspicion of dementia.

### Pure-Tone Audiometry

**FIGURE 3:**
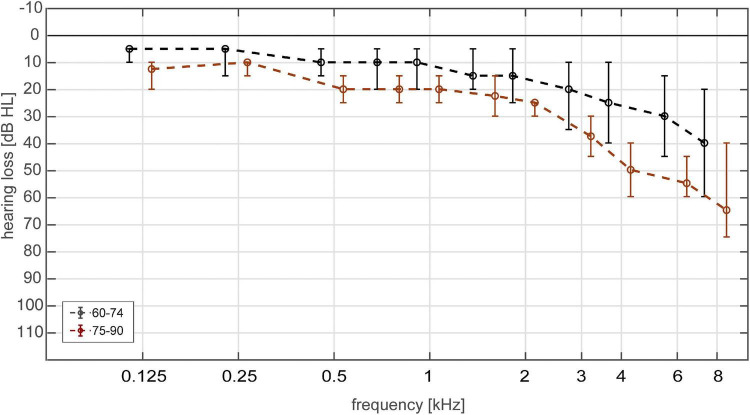
Hearing thresholds (median and interquartile range) of 80 ears (40 subjects) divided into two age groups. Age groups were 60–74 (black) and 75–90 (red) years.

Pure-tone thresholds of the 40 subjects (for better illustration divided into two age groups: 60–74, 75–90 years) are shown in Figure 3. According to the classification of hearing loss published by the World Health Organization [WHO] (2001) 26 subjects had normal hearing in both ears and three subjects had normal hearing in one ear. Seven subjects had a mild hearing loss in both ears and five subjects in one ear. One subject had a moderate hearing loss in both ears and four subjects in one ear. None of the subjects had a severe/profound hearing loss or deafness in any ear.

Median PTA_4 was 18.75 dB HL (IQR: 12.5-27.5 dB HL), median PTA_high was 35 dB HL (IQR: 20-56.7 dB HL).

### Speech Perception in Noise

**FIGURE 4:**
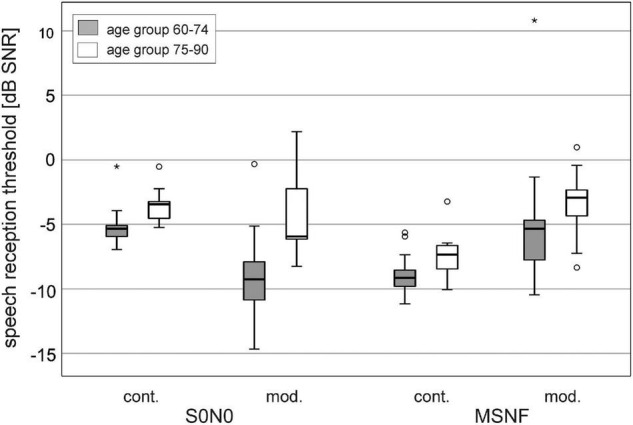
Speech reception thresholds in spatial conditions S0N0 and multi-source noise field (MSNF) for the continuous (cont.) noise and modulated (mod.) noise divided into age groups 60–74 years (gray boxes) and 75–90 years (white boxes). * Outliers 3 times greater than interquartile range.

The SRT results divided in two age groups 60–74 years and 75–90 years for the different test conditions are shown in Figure 4. The age group 60–74 years showed significantly lower mean SRTs than the older age group in all test conditions (S0N0 continuous noise: Z = −3.715, *p* < 0.001; S0N0 modulated noise: Z = −3.515, *p* < 0.001; MSNF continuous noise: Z = −2.642, *p* = 0.016; MSNF modulated noise: Z = −2.236, *p* = 0.025).

**FIGURE 5:**
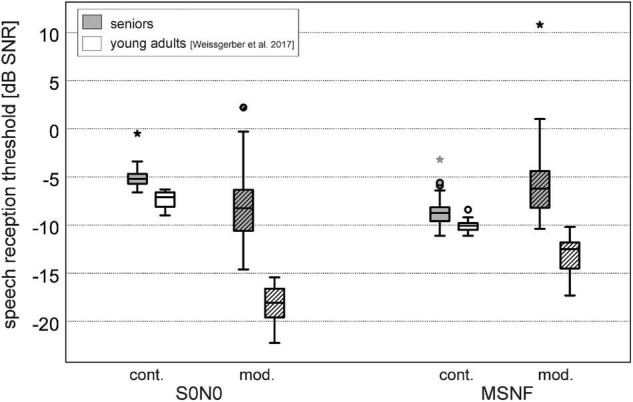
Speech reception thresholds for the continuous noise (cont., filled boxes) and modulated noise (mod., hatched boxes) in spatial conditions S0N0 and multi-source noise field (MSNF). Gray boxes: seniors (present study), white boxes: data of young adults with normal hearing (*n* = 14, mean age: 26.4 ± 5.4 years, range: 22–37.3 years) using the identical test setup (Weissgerber et al., 2017). * Outliers 3 times greater than interquartile range.

Boxplots of SRT results averaged over all older study participants were shown in Figure 5 (gray boxes). The median SRT in S0N0 condition using continuous noise was −5.2 dB SNR (IQR: −4.5 to −5.8 dB SNR). Median SRT in S0N0 with modulated noise was significantly better than in continuous noise (difference: 3.1 dB, Z = −5.216, *p* < 0.001). The IQR of the SRTs obtained in modulated noise (−6.2 to −10.6 dB SNR) was 3.1 dB larger than the IQR for continuous noise.

The SRT in MSNF using continuous noise was −8.8 dB SNR (IQR: −8.1 to −9.7 dB SNR), 3.7 dB significantly lower than the SRT for the modulated noise (Z = −5.276, *p* < 0.001). The IQR of the SRTs obtained in MSNF with modulated noise (−3.0 to −7.4 dB SNR) was 2.8 dB larger than the IQR for continuous noise.

Speech reception thresholds with continuous noise were significantly higher in the S0N0 condition than in the MSNF condition (3.6 dB difference, Z = −5.513, *p* < 0.001). In the modulated noise condition, a significantly lower SRT was obtained in S0N0 condition compared with MSNF (3.2 dB difference, Z = −4.678, *p* < 0.001).

#### Comparison With Young Adults

Speech reception thresholds results obtained in young adults with normal hearing using the identical test setup (Weissgerber et al., 2017) are illustrated in Figure 5 (white boxes). SRTs in the young normal hearing group were significantly lower for all test conditions (*p* < 0.001). Mean difference was lowest for test conditions using continuous noise (S0N0: 1.9 dB; MSNF: 1.3 dB). More important, in conditions with modulated noise large differences between younger and older subjects were found (S0N0: 9.8 dB; MSNF: 6.3 dB).

### Sound Localization

The median relative localization error was 5.8° (interquartile range: 4.5–8.1°) and the median amount of front-back confusions was 12.9% (interquartile range: 5.0–31.1%).

Results of mean localization error and front-back confusions divided in two age groups 60–74 years and 75–90 years are provided as Supplementary Material. There was no significant difference between the age group 60–74 years and the age group 75–90 years for both measures localization error and front-back confusions. There was also no significant difference between the localization error of the seniors measured in this study and a young group with normal hearing (*n* = 9, mean age: 30.3 ± 6.1 years).

**FIGURE 6:**
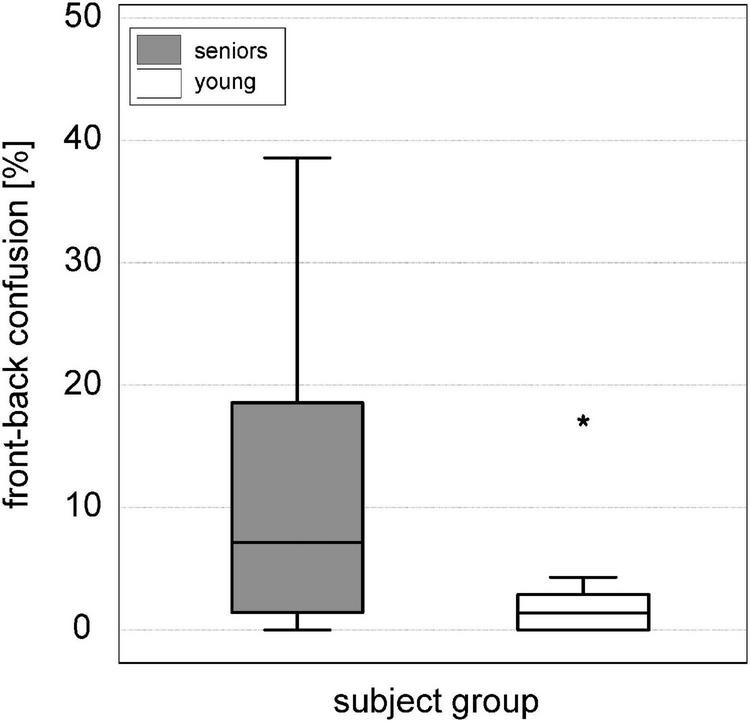
Boxplots of front-back confusions [%] in the sound localization test for the senior study participants (gray box) compared to a reference group of nine young adults with normal hearing (white box). * Outliers 3 times greater than interquartile range.

The results of front/back confusions are shown in Figure 6. Additionally, reference data obtained in young adults with normal hearing using the identical test setup is illustrated. The amount of front-back confusions was significantly worse in the test subjects of the present study (difference: 11.5%; Z = −3.213, *p* < 0.001) compared with young adults.

### Correlations With Age

Correlations in the senior group between age and DemTect scores, pure-tone hearing thresholds, and speech in noise scores and were shown in Table 1. Additionally, correlations were calculated after partialling out mean high-frequency hearing loss (PTA_high).

**TABLE 1:** Spearman correlation coefficients ρ for results on measures of DemTect, hearing loss, and speech perception in noise vs. age (first column).

	Age	Age PTA_high partialled out
DemTect	**−0.412**	−0.323
PTA_4	**0.367**	0.147
PTA_high	**0.353**	N/A
SRT S0N0 cont.	**0.539**	**0.44**
SRT S0N0 mod.	**0.426**	0.286
SRT MSNF cont.	**0.383**	0.289
SRT MSNF mod.	**0.398**	0.288

*Correlation coefficients after partialling out high-frequency hearing loss PTA_high (second column). Gray values indicate non-significant correlations (p > 0.05). Values in black indicate significant results (p ≤ 0.05). Values in boldface indicate significant results after applying a Bonferroni-Holm correction. N/A: correlation non-applicable.*

A significant negative correlation between age and DemTect score was found (ρ = −0.412, *p* = 0.04). There was significant correlation between age and PTA_4 (ρ = 0.367, *p* = 0.045) as well as between age and PTA_high (ρ = 0.353, *p* = 0.045). There was a significant correlation between age and S0N0 SRT for both types of noise (continuous: ρ = 0.539, *p* < 0.001, modulated: ρ = 0.426, *p* = 0.036). There was a significant correlation between age and MSNF SRT for continuous noise (ρ = 0.383, *p* = 0.045) and modulated noise (ρ = 0.398, *p* = 0.044).

After partialling out high-frequency hearing loss, only a significant correlation between age and S0N0 SRT in continuous noise (ρ = 0.44, *p* = 0.03) was found.

### Correlations With Cognitive Performance

Correlations in the senior group between DemTect scores and both measures pure-tone hearing thresholds and speech in noise scores were shown in Table 2. Correlations were also calculated after partialling out mean high-frequency hearing loss (PTA_high).

**TABLE 2:** Spearman correlation coefficients ρ for results on measures of hearing loss and speech perception in noise vs. DemTect score (first column).

	DemTect	DemTect PTA_high partialled out
PTA_4	−0.284	0.036
PTA_high	–0.375	N/A
SRT S0N0 cont.	**−0.461**	–0.325
SRT S0N0 mod.	–0.382	−0.212
SRT MSNF cont.	−0.203	−0.072
SRT MSNF mod.	**−0.502**	–0.404

*Correlation coefficients after partialling out high-frequency hearing loss PTA_high (second column). Gray values indicate non-significant correlations (p > 0.05). Values in black indicate significant results (p ≤ 0.05). Values in boldface indicate significant results after applying a Bonferroni-Holm correction. N/A: correlation non-applicable.*

There was a significant negative correlation between the DemTect and the S0N0 SRT in continuous noise (ρ = −0.461, *p* = 0.015) and between the DemTect score and the MSNF SRT in modulated noise (ρ = −0.502, *p* = 0.006). A scatterplot showing individual DemTect scores and MSNF SRTs in modulated noise is provided as Supplementary Material. Furthermore, a negative correlation between PTA_high and DemTect score (ρ = −0.375, unadjusted *p* = 0.017) and between S0N0 SRT in modulated noise and DemTect score (ρ = −0.382, unadjusted *p* = 0.015) was found which failed to reach significance after Bonferroni-Holm correction (*p* = 0.06 for both correlation coefficients).

After partialling out high-frequency hearing loss, no significant correlations between DemTect score and all other measures were found.

### Subjective Hearing Performance

**FIGURE 7:**
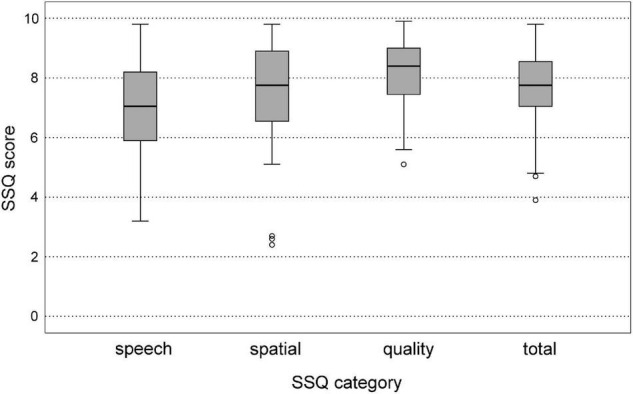
Boxplots of speech, spatial, and qualities of hearing scale (SSQ) scores for the three subsets speech, spatial, quality, and for the mean total SSQ score.

In the evaluation of the SSQ questionnaire, both the obtained scores of the three sections of the questionnaire individually and the score of the whole questionnaire were analyzed. Boxplots of the SSQ results are shown in Figure 7. In the subjective evaluation of speech perception (SSQ section 1) the median score was 7.1 points (IQR: 5.9–8.4 points). The median of the subjective evaluation of spatial hearing (SSQ section 2) was 7.8 points (IQR: 6.5–9.0 points). The median score increased to 8.4 points (IQR: 7.4–9.1 points) for the subjective rating of hearing quality (SSQ section 3). The median score of the complete SSQ questionnaire was 7.8 points (IQR: 7.0–8.6 points).

Correlations between age and SSQ scores are shown in Table 3. There was no correlation between age and SSQ scores for speech and spatial perception in the senior study population. A correlation between on one hand age and on the other SSQ scores for sound quality (ρ = −0.364, unadjusted *p* = 0.021) and the total SSQ scores (ρ = −0.326, unadjusted *p* = 0.039) failed to reach significance after Bonferroni-Holm correction (*p* = 0.084 and *p* = 0.117). After partialling out high-frequency hearing loss, no significant correlations between age and SSQ scores were found.

**TABLE 3:** Spearman correlation coefficients ρ for results on SSQ scores (speech, spatial, quality, and total SSQ score) vs. age (first column).

	Age	Age PTA_high partialled out
SSQ speech	−0.23	−0.113
SSQ spatial	−0.299	−0.246
SSQ quality	−0.364	−0.295
SSQ total	−0.326	−0.235

*Correlation coefficients after partialling out high-frequency hearing loss PTA_high (second column). Gray values indicate non-significant correlations (p > 0.05). Values in black indicate significant results (p ≤ 0.05). After applying a Bonferroni-Holm correction no significant correlations were found.*

#### Correlation Between Subjective Hearing and Test Results

Spearman rank correlations were calculated between SSQ speech scores of the senior subject group and high-frequency hearing loss and SRTs in noise. SSQ spatial scores were analyzed for potential correlations with high-frequency hearing loss, SRTs in spatially separated noise and localization scores. Furthermore, correlations between mean total SSQ score and all hearing performance measures were calculated. The correlation coefficients are shown in Table 4.

**TABLE 4:** Spearman correlation coefficients ρ for results on measures of hearing loss, speech perception in noise, and sound localization ability vs. SSQ scores of speech perception (first column), spatial hearing (second column) and total SSQ score (third column).

	SSQ speech	SSQ spatial	SSQ total
PTA_high	–0.373	−0.209	–0.343
SRT S0N0 cont.	**−0.433**	−	**−0.482**
SRT S0N0 mod.	–0.384	−	–0.403
SRT MSNF cont.	−0.285	−0.105	−0.242
SRT MSNF mod.	–0.388	−0.283	–0.357
localization error	−	−0.32	–0.316
front-back confusion	−	−0.145	−0.186

*SSQ scores of speech perception were correlated with PTA_high and all SRTs in noise. SSQ scores of spatial perception were correlated with PTA_high, SRTs in MSNF, and localization measures. Gray values indicate non-significant correlations (p > 0.05). Values in black indicate significant results (p ≤ 0.05). Values in boldface indicate significant results after applying a Bonferroni-Holm correction. −: no correlations were calculated.*

A significant negative correlation (after applying a Bonferroni-Holm correction) was found between the SSQ score of speech perception and the SRT in configuration S0N0 for continuous noise (ρ = −0.433, *p* = 0.025). Negative correlations between SSQ score of speech and PTA_high (ρ = −0.373, unadjusted *p* = 0.018) and SRTs in modulated noise (S0N0: ρ = −0.384, unadjusted *p* = 0.014; MSNF: ρ = −0.388, unadjusted *p* = 0.013) failed to reach significance after applying a Bonferroni-Holm correction (*p* = 0.052 for all three correlations).

A negative correlation between the SSQ score for spatial hearing and localization error (ρ = −0.32, unadjusted *p* = 0.044) failed to reach significance after applying a Bonferroni-Holm correction (*p* = 0.22).

There was a significant negative correlation between the mean total SSQ score and the SRT in the configuration S0N0 in continuous noise (ρ = −0.482, *p* = 0.014). Negative correlations between total SSQ score and PTA_high (ρ = −0.343, unadjusted *p* = 0.03), SRTs in modulated noise (S0N0: ρ = −0.403, unadjusted *p* = 0.01; MSNF: ρ = −0.357, unadjusted *p* = 0.024) and localization error (ρ = −0.316, unadjusted *p* = 0.047) became non-significant (*p* = 0.12/0.06/0.12/0.141) after correction for multiple comparisons.

## Discussion

### Speech Perception in Noise

In the senior subject group a significant impact of age on speech perception was found for both noise types and spatial noise configurations. Highest differences to young normal hearing adults were found in conditions with modulated noise. Furthermore, subjects with lower cognitive scores showed higher SRTs for both noise types in co-located target and masker condition and for modulated noise in the spatial masker condition MSNF. It should be noted that many of the senior subjects had age-related hearing loss. Therefore, especially high-frequency hearing loss was a confounding factor in this subjects which presumably accounted more for the decline in speech in noise as age itself. Accordingly, only one out of the four speech tests in noise correlated with age after partialling high-frequency hearing loss out. Still, even in the present cohort of seniors with mainly mild hearing loss, speech perception in certain situations of daily life (depending on signal-to-noise ratio) could be substantially degraded as reflected in the speech in noise tests using MSNF. The slope of the speech discrimination function for modulated noise in S0N0 and MSNF is ~6%/dB. Therefore, mean deterioration of speech perception in modulated noise in compared with young adults was 58.8% (S0N0) and more than 37 percent (MSNF). In conditions using continuous noise differences of about 18% (MSNF) and 31% (S0N0) were found. Since speech perception in clinical routine is mainly assessed in quiet or in continuous noise, it could be expected that deficits in speech perception in the elderly in everyday conditions are oftentimes underestimated.

In the co-located target and masker condition S0N0, mean SRTs in modulated noise were significantly lower than in continuous noise. The amplitude-modulated noise contains temporal gaps, which enables for release from masking (RM). Furthermore, Stone et al. (2011, 2012) showed that the inherent fluctuations in “continuous” noise also have a masking effect which leads to worse SRTs compared to temporally modulated noise with low modulation rates.

In our study RM was found to be 3.1 dB and, thus, 7.9 dB poorer than in a normal hearing adult group. A possible explanation for the reduced RM in seniors in the present study could be that these seniors had age-related hearing loss. As one result, audibility for sibilants is reduced. There are also deficits in frequency selection resulting in poorer separation of speech and noise and making temporal gaps more difficult or impossible to detect (Moore, 1985). Duquesnoy (1983) also found that older persons aged 75–88 years with presbycusis are less able to use the temporal gaps in fluctuating noise than normal-hearing persons. Peters et al. (1998) showed that both age and hearing impairment have a considerable influence on speech perception with highest impact in modulated noise. For young adults with normal hearing, RM was up to 4-7 dB whereas in older persons with hearing loss only 1.5 dB improvement was documented. Results from van Summers and Molis (2004) imply that signal audibility is not the major factor limiting RM in the presence of a mild to moderate hearing impairment. Hearing loss reduced the benefit from masker fluctuations for the majority of their study subjects even for an increase in presentation level of up to 30 dB. Rather, distortions in the processing of suprathreshold speech may account for reduced RM. Another aspect is a potential deterioration of temporal resolution. Results by Füllgrabe (2013) showed that temporal processing is reduced with increasing age even in the absence of a peripheral hearing loss. Sensitivity to temporal fine structure decreased in a monaural as well as binaural task with increasing age already beginning in early midlife.

The role of binaural processing is evident in the tested MSNF condition where speech and noise were spatially separated. Spatial release from masking (SRM) leads to improved SRTs compared to a co-located masker and target position due to the head shadow and binaural squelch effect. In the study by Duquesnoy (1983) it was found that binaural listening (noise signal from side, speech signal from front) could improve SRTs by 5–9 dB for young normal-hearing persons and by only 3–4 dB for elderly persons with age-related hearing loss. In the present study, SRTs for continuous noise were also significantly lower in MSNF than in S0N0 condition in elderly subjects. SRM in continuous noise was found to be in the same range than for young normal hearing adults. In the MSNF condition with four uncorrelated modulated noise sources the effect monaural unmasking is reduced compared to co-located speech and masker presentation. Even though temporal gaps are smaller than in single noise co-located masker condition, young normal hearing subjects still show 2.4 dB better SRTs than in MSNF with continuous noise (i.e., combined effects of monaural release from masking and SRM). On the other hand, MSNF SRTs in the senior group were 2.6 dB worse for modulated noise than for continuous noise in spite of the presence of temporal gaps. Thus, SRTs were even worse compared to continuous noise. This effect could be caused by distortions of binaural temporal processing in the senior subject group. A review on the relationship between hearing loss and age and binaural processing is given by Moore (2021). Füllgrabe and Moore (2018) conducted a meta-analysis on the relations between binaural temporal fine structure sensitivity and hearing loss and age. Hearing loss and age were significantly negatively correlated to temporal fine structure sensitivity where age was a better predictor than audiometric threshold. Reduced temporal binaural processing could not solely explain the disruptive effect of modulated noise on SRM in MSNF. It is conceivable that in demanding binaural test conditions cognitive performance is more influential on auditory performance than in co-located masker conditions.

In the present study, DemTect scores correlated significantly with two out of four OLSA SRTs. During the OLSA task the subject has to remember five words before recalling them on a touch screen display. Working memory stores verbal information while processing that information (Baddeley and Hitch, 1974). If the verbal information matches information in the mental lexicon of long-term memory, the speech signal is recognized and processed. Hwang et al. (2017) showed that age and working memory capacity influences speech perception in noise in a group of hearing impaired subjects aged 24–80 years whereas no effect of age and only a low effect of working memory (subtest digit backward span) was found in a normal hearing group aged 27–73 years. Rudner et al. (2011) reported that hearing impaired subjects with higher working memory capacity performed better in speech perception in modulated noise. Deficits in cognitive performance could lead to an increased listening effort and may reduce the use of the temporal gaps for RM.

### Sound Localization

The mean localization error in our study population was only slightly but not significantly increased compared with normal hearing young adults. This is in line with results reported by Otte et al. (2013) who measured sound localization in a group of normal hearing young adults (20–34 years) and older adults (63–80 years) with mild to moderate high-frequency hearing loss (mean hearing thresholds of subject group). However, the literature also contains studies showing that the ability to localize sound decreases with age. In such studies oftentimes localization ability was not only assessed for broad-band noise but also for narrow-band or highpass-filtered and lowpass-filtered noise stimuli (Abel et al., 2000; Dobreva et al., 2011; Freigang et al., 2015). Freigang et al. (2015) reported a decrease in sound localization accuracy for older adults which was most prominent for lateral sound source positions and high-frequency stimuli. Dobreva et al. (2011) reported a decrease in sound localization ability in the horizontal plane with age (for subjects groups aged 45–66 and 70–81 years in comparison to younger adults aged 19–41) for both broadband noise and narrowband noise. The discrepancy in the results might be due to differences in the methodology for measuring sound localization ability (e.g., angular span, pointer method, stimulus type and presentation level, amount of level roving, etc.) and in the distribution of age and hearing loss of the test subjects. The difference in mean localization error for broadband noise between younger and older subjects reported in the present study could be considered as clinically irrelevant. However, it cannot be ruled out that even in our subject group localization ability for higher frequency narrow-band sounds is deteriorated.

This is supported by the result of a significantly higher amount of front-back confusions compared with young adults which was confirmed in two other studies (Abel et al., 2000; Otte et al., 2013). The occurrence of front-back confusions seem to be directly related to the high-frequency hearing loss of elderly subjects. Interaural time differences and interaural level differences are the dominant cues for horizontal sound localization, whereas high-frequency monaural cues contribute significantly to vertical sound localization as well as to resolving front-back confusions. It was hypothesized that poor coding of interaural time differences in older subjects with presbycusis accounts for deficits in sound localization ability in the horizontal plane (Dobreva et al., 2011).

In the present study no significant correlation between the results of cognitive performance and the localization ability of sounds was found. Likewise, Neher et al. (2011) reported no cognitive measures as predictors for sound localization ability.

### Cognition and Hearing Loss

Sixty-five percent of the subjects showed age-appropriate cognitive performance in the DemTect test, and 35% of the subjects showed a mild cognitive impairment. None of the subjects were suspected of having dementia. Since study subjects were recruited for participation via flyers and an advertisement at the grounds of the University Hospital Frankfurt, it is also conceivable that seniors with reduced cognitive performance who were already being cared for by nursing at home or in a retirement home may not have been reached at all. Therefore, our study group cannot be considered as representative.

Memory span of the working memory decreases with age (Cattell, 1971). This was also shown in the Berlin Aging Study (Lindenberger et al., 2010). In order to determine cognitive performance, 14 cognitive tests were performed, which could be assigned to five cognitive abilities. All five abilities were shown to decrease linearly with age, especially those abilities that belong to fluid intelligence. In our subject group without any severe cognitive impairment the cognitive-test performance also correlated significantly with age. This is surprising since the scores of the DemTect test are age-corrected.

On the other hand, the correlation between age and DemTect score vanished after partialling out high-frequency hearing loss and applying Bonferroni-Holm correction for multiple comparisons. It was also shown that subjects with higher age and more severe hearing loss (i.e., higher PTA_high) and/or with higher SRTs in noise (three out of four tests) tended to have lower DemTect scores. The Berlin Aging Study also reported that individuals with poorer hearing also had poorer cognitive performance (correlation *r* = 0.5). In a study by Lin et al. (2013) it was even observed that hearing loss can lead to an accelerated decline in cognitive performance by up to 30–40%. Study participants with hearing loss who did not wear hearing aids had slightly worse scores on cognitive tests than study participants with hearing aids.

However, it must also be considered that misunderstandings in verbal communication during the test procedure due to hearing loss could also impair test scores in the assessment of cognitive abilities. Füllgrabe (2020) showed that young participants with simulated hearing loss performed significantly worse in cognitive tasks using acoustically presented test items (forward digit span, backward digit span, listening span) than a control group with the same age without hearing loss. It was concluded that cognitive impairments could be overestimated in the presence of a hearing impairment.

Castiglione et al. (2019) introduced an audiological screening model of subjects at risk of cognitive decline with slight to moderate hearing loss. It could potentially be useful to screen elderly subjects with hearing loss for dementia on regular basis and to conduct hearing test in patients suffering from an onset of dementia.

### Subjective Hearing Performance

The present study included subjects who described themselves as having normal hearing and who did not use hearing aids. However, only 26 of the 40 subjects had normal hearing in both ears and three subjects had normal hearing in one ear. In five of the 40 test persons, even the indication for a unilateral hearing aid provision was present, and five other test persons even had an indication for bilateral hearing aid provision.

Considering the correlations of SSQ scores with high-frequency pure-tone hearing loss and speech perception in noise (S0N0 in continuous noise) there was at least some awareness of the subjects for an own auditory deficit. Nevertheless, all of them described themselves as having no hearing problems. This suggests that not only the own perception of hearing loss is a problem for seniors, but also to accept hearing disabilities and deciding to seek help is a challenge. Carson (2016) reports that it takes an average of 7–10 years for a person to seek medical help after the first recognized signs of hearing loss. Since presbycusis is an age-related condition, its acceptance also means acceptance of aging, which for some seniors may mean a reduction in independence or a loss of control. According to Donahue et al. (2010) only one in five people suffering from age-related hearing loss sought professional help. Therefore, it would be advisable that seniors should undergo routine hearing screenings in order to detect hearing disorders as soon as possible, so that hearing aids can be prescribed at an early stage.

### Potential Limitations of the Study

All subjects without self-reported hearing complaints were included. Since subjects suffering from so far unnoticed hearing loss were not excluded the study population is partly inhomogeneous. Another drawback is potentially that subjects with asymmetric hearing loss were included. A higher amount of subjects aged 80 and older would be desirable to extent the quality of correlation analysis. Furthermore, additional tests on temporal processing (e.g., on the perception of temporal fine structure) are in need to interpret deficits in speech perception in modulated noise or spatial noise conditions and its relation to cognitive performance.

## Conclusion

Although no complaints about hearing ability were reported in the present study group of seniors, the results of the study support the hypotheses that hearing performance decreases with increasing age together with declining cognitive abilities even if not detected by the subject itself. This holds especially for speech perception in noise in complex conditions where intact binaural hearing is a mandatory requirement. Therefore, special attention should be given to hearing screening programs to improve the quality of life of older people. Speech perception tests using temporally modulated noise can serve as a screening method for early detection of hearing disorders in older adults. Hearing screening should also be mandatory for dementia patients, just as dementia screening is mandatory for seniors with known hearing loss. Further research is needed to investigate on the causality between dementia and hearing loss.

## Data Availability Statement

The raw data supporting the conclusions of this article will be made available by the authors, without undue reservation.

## Ethics Statement

The studies involving human participants were reviewed and approved by Ethics Committee of the Department of Medicine of the Goethe-University Frankfurt am Main, Germany (No. 164/13). The patients/participants provided their written informed consent to participate in this study.

## Author Contributions

TW and UB contributed to the conception and design of the study. CM and TW organized and conducted the experiments, performed the statistical analysis, and wrote the first draft of the manuscript. All authors contributed to the interpretation of the data, manuscript revision, and read and approved the submitted version.

## Conflict of Interest

The authors declare that the research was conducted in the absence of any commercial or financial relationships that could be construed as a potential conflict of interest.

## Publisher’s Note

All claims expressed in this article are solely those of the authors and do not necessarily represent those of their affiliated organizations, or those of the publisher, the editors and the reviewers. Any product that may be evaluated in this article, or claim that may be made by its manufacturer, is not guaranteed or endorsed by the publisher.
