# Supplementary material for: Age Differences in Speech Perception in Noise and Sound Localization in Individuals With Subjective Normal Hearing
Source: Front Psychol. 2022 Apr 15;13:845285. doi: 10.3389/fpsyg.2022.845285 (PMC9051364; doi:10.3389/fpsyg.2022.845285)
Supplement: Supplementary file 1 [file Image_1.PDF]

Supplementary Material

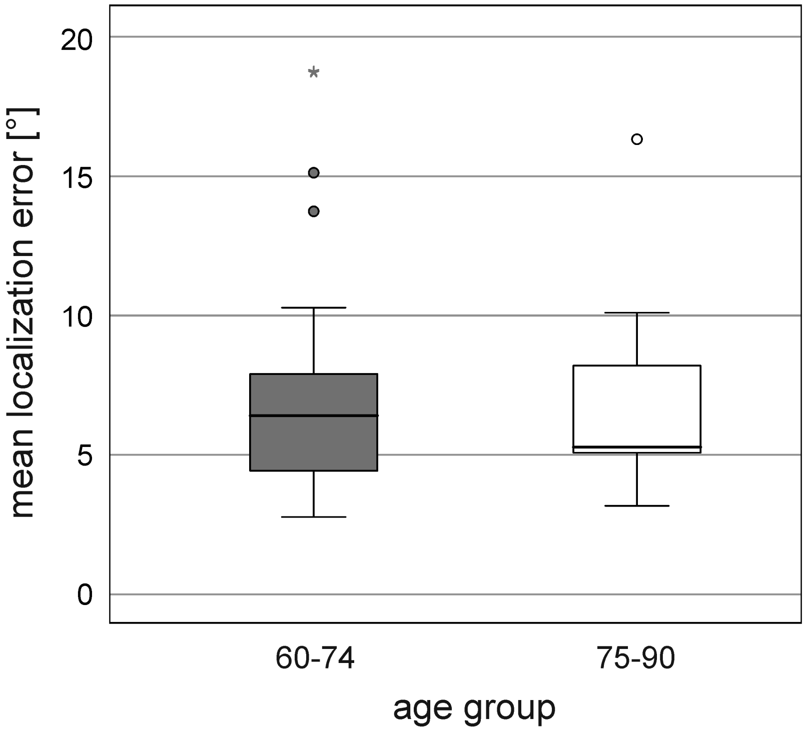

Supplementary Figure 1. Boxplots of mean localization error [°] in the sound localization test divided into age groups 60-74 years (grey box) and 75-90 years (white box).

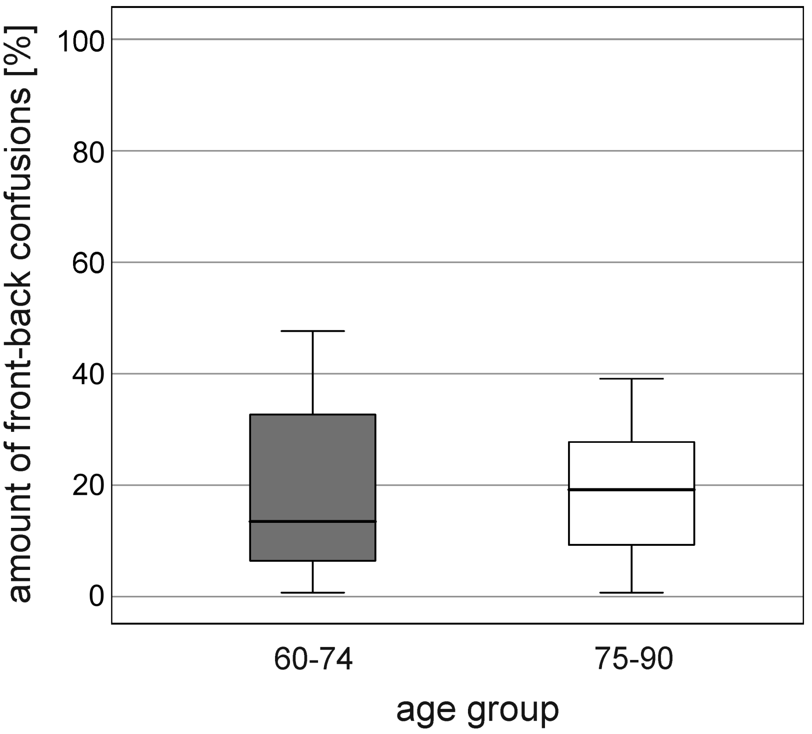

Supplementary Figure 2. Boxplots of front-back confusions [%] in the sound localization test divided into age groups 60-74 years (grey box) and 75-90 years (white box).

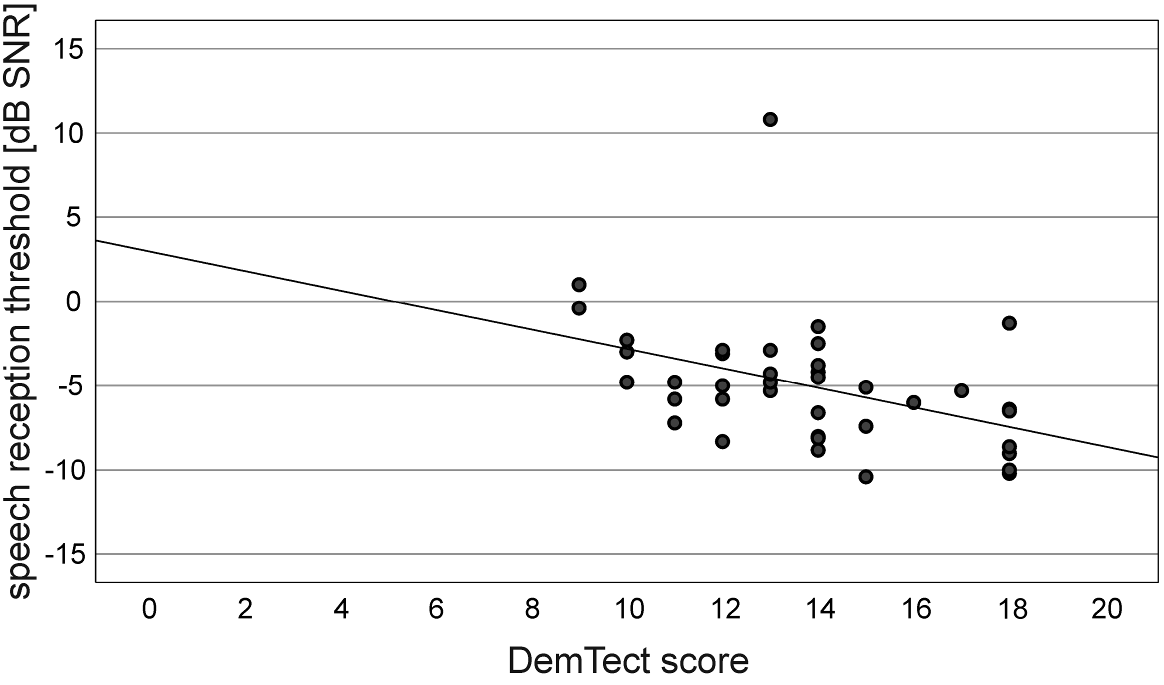

Supplementary Figure 3. Scatter plot of DemTect scores and speech reception thresholds [dB SNR] obtained in MSNF with modulated noise.
